# Supplementary material for: Time-travelling pathogens and their risk to ecological communities
Source: PLoS Comput Biol. 2023 Jul 27;19(7):e1011268. doi: 10.1371/journal.pcbi.1011268 (PMC10374110; doi:10.1371/journal.pcbi.1011268)
Supplement: S1 Table — Invasion outcomes are measured in terms of persistence of invaders and their descendants in the invaded communities, and relative changes in diversity in the invasion simulations compared to control simulations. (DOCX) [file pcbi.1011268.s001.docx]

|  | **invader persistence** | | **diversity change** | | **absolute diversity change** | |
| --- | --- | --- | --- | --- | --- | --- |
|  | ***r_s_*** | ***p*** | ***r_s_*** | ***p*** | ***r_s_*** | ***p*** |
| ***Invader features*** |  |  |  |  |  |  |
| *world size* | 0.12 | 0.001 | -0.08 | 0.0259 | -0.04 | 0.2358 |
| *resource availability* | 0.03 | 0.3417 | -0.03 | 0.4546 | 0.05 | 0.217 |
| *resource diversity* | 0.01 | 0.8216 | -0.03 | 0.4637 | 0.07 | 0.0729 |
| *task diversity* | 0.56 | <0.0001 | -0.10 | 0.0045 | 0.09 | 0.0117 |
| *max task complexity* | 0.56 | <0.0001 | -0.14 | 0.0002 | 0.09 | 0.0158 |
| *pre-invasion mean abundance* | 0.48 | <0.0001 | -0.10 | 0.0090 | 0.07 | 0.0655 |
| *pre-invasion total abundance* | 0.47 | <0.0001 | -0.09 | 0.0195 | 0.05 | 0.2017 |
| *abundance in source community* | 0.45 | <0.0001 | -0.07 | 0.0585 | 0.05 | 0.1571 |
| *pre-invasion persistence* | 0.39 | <0.0001 | -0.06 | 0.0797 | 0.02 | 0.6526 |
| *generalism (source community)* | 0.24 | <0.0001 | 0.04 | 0.3053 | -0.05 | 0.2028 |
| *generalism (invaded community)* | 0.70 | <0.0001 | -0.07 | 0.0669 | 0.08 | 0.0364 |
| *evolutionary age* | 0.29 | <0.0001 | -0.07 | 0.0738 | 0.07 | 0.0726 |
| ***Features of the invaded community*** |  |  |  |  |  |  |
| *free-living abundance* | -0.04 | 0.3369 | -0.09 | 0.0191 | -0.16 | <0.0001 |
| *free-living richness* | -0.06 | 0.1054 | -0.08 | 0.0306 | -0.16 | <0.0001 |
| *pathogen richness* | -0.14 | 0.0001 | 0.00 | 0.9415 | -0.33 | <0.0001 |
| *average phylogenetic distance* | -0.22 | <0.0001 | 0.09 | 0.0126 | -0.11 | 0.0025 |
| *pathogen abundance* | -0.24 | <0.0001 | 0.01 | 0.7746 | -0.34 | <0.0001 |
| *free-living density* | -0.25 | <0.0001 | -0.05 | 0.1743 | -0.22 | <0.0001 |
| *pathogen density* | -0.36 | <0.0001 | 0.05 | 0.1334 | -0.34 | <0.0001 |
| ***Others*** |  |  |  |  |  |  |
| *invasion time* | -0.04 | 0.2447 | 0.00 | 0.9999 | 0.08 | 0.0364 |
| *time difference* | -0.28 | <0.0001 | 0.05 | 0.1671 | -0.03 | 0.4708 |
