# Supplementary figures and images for: Time-travelling pathogens and their risk to ecological communities
Source: PLoS Comput Biol. 2023 Jul 27;19(7):e1011268. doi: 10.1371/journal.pcbi.1011268 (PMC10374110; doi:10.1371/journal.pcbi.1011268)

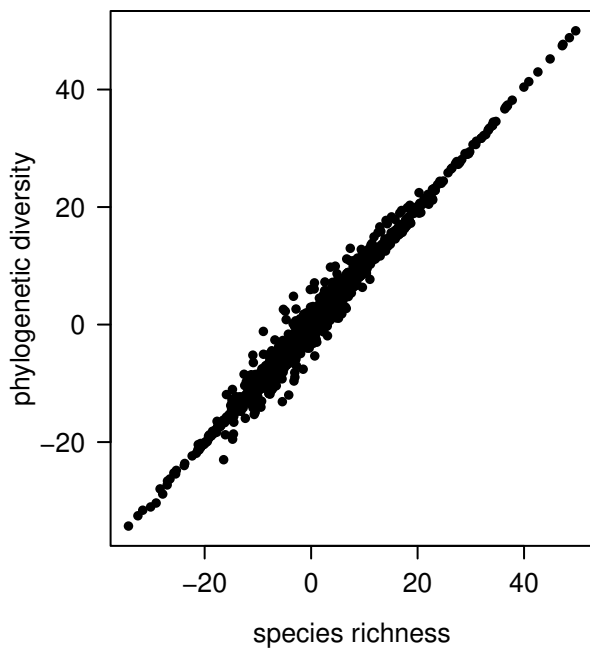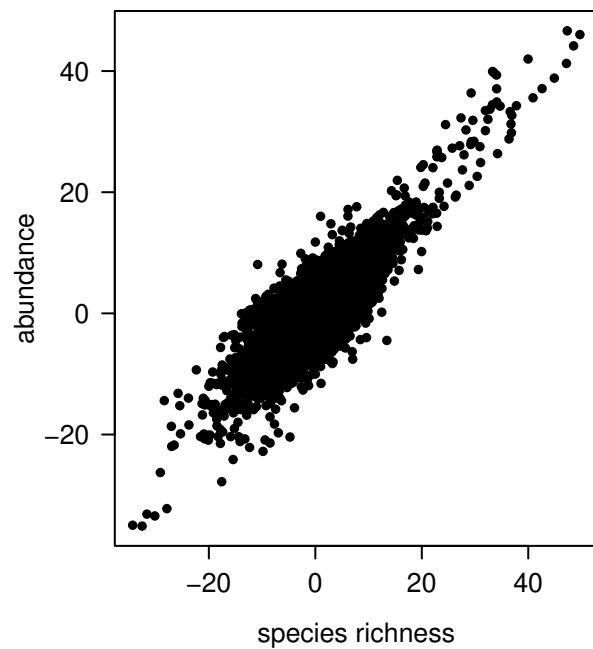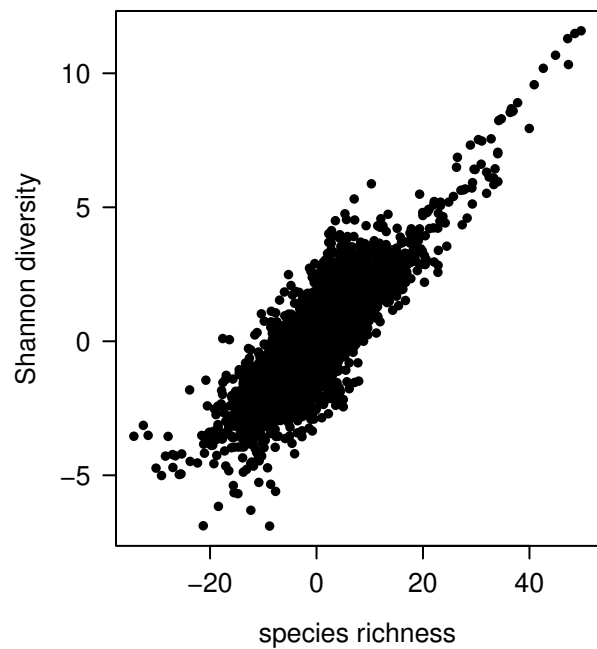

Supplement: S1 Fig — (PDF) [file pcbi.1011268.s002.pdf]

**a**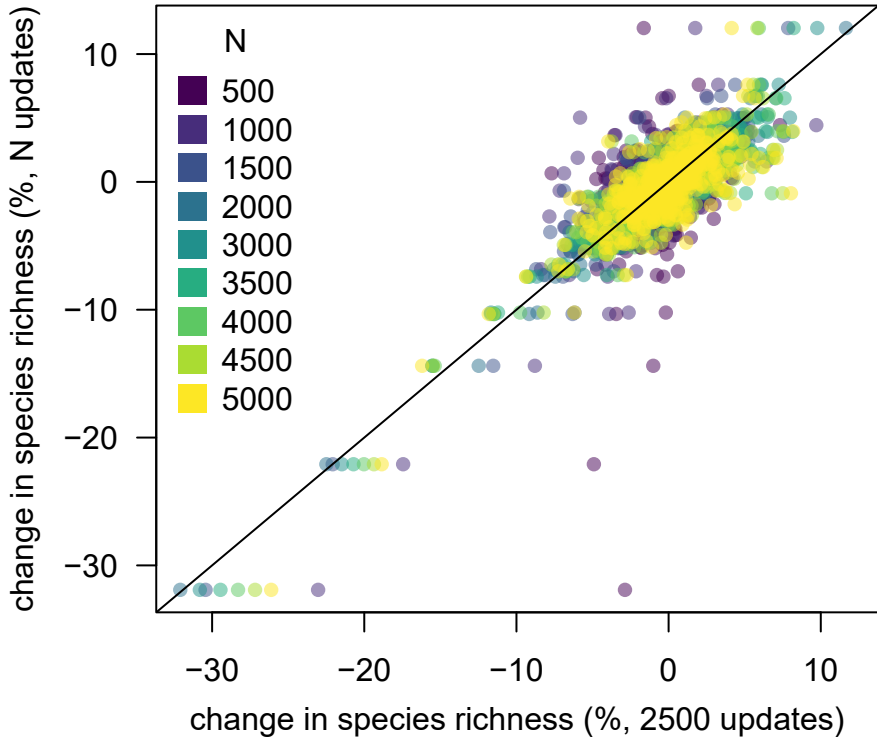**b**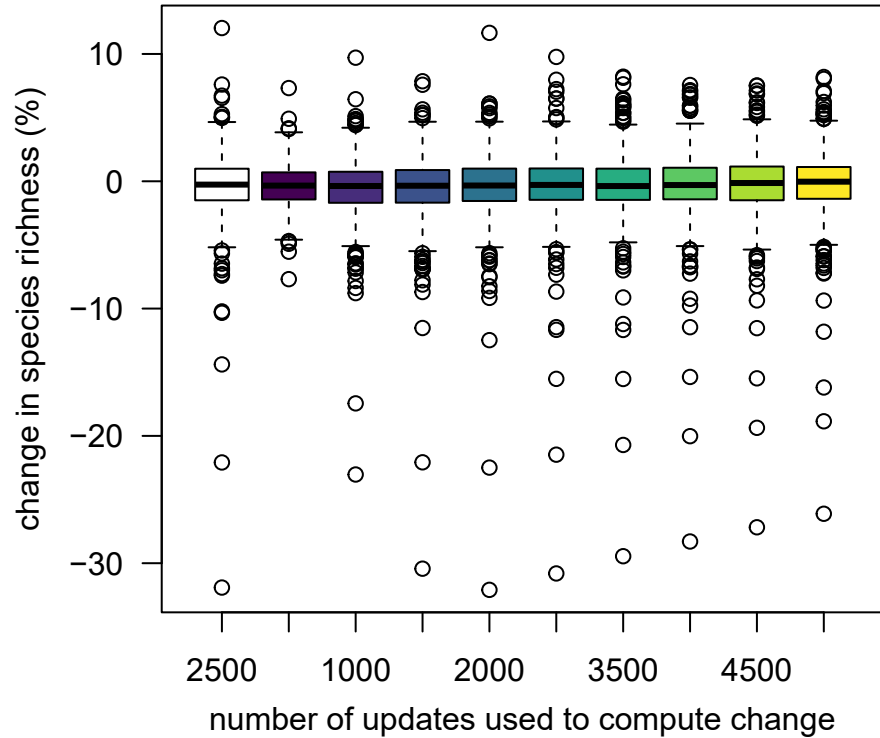

Supplement: S2 Fig — (a) Relative post-invasion % change in free-living diversity in control versus invasion simulations computed in a time window of 2500 updates (x axis), compared to the corresponding values obtained using alternative time windows spanning 500 to 5000 updates (y axis, different colours). (b) Boxplots summarizing the relative post-invasion % change in free-living diversity in control versus invasion simulations computed using alternative time-windows spanning 500 to 5000 updates (the first, white boxplot refers to the actual time window we used in the analyses, i.e., 2500 updates, corresponding to 1% of the total number of updates in a simulation). Boxes indicate 1st and 3rd quartiles, horizontal lines indicate median values, whiskers indicate largest/lowest points inside the range defined by the 1st or 3rd quartile + 1.5 times the interquartile range and circles indicate outliers. (PDF) [file pcbi.1011268.s003.pdf]

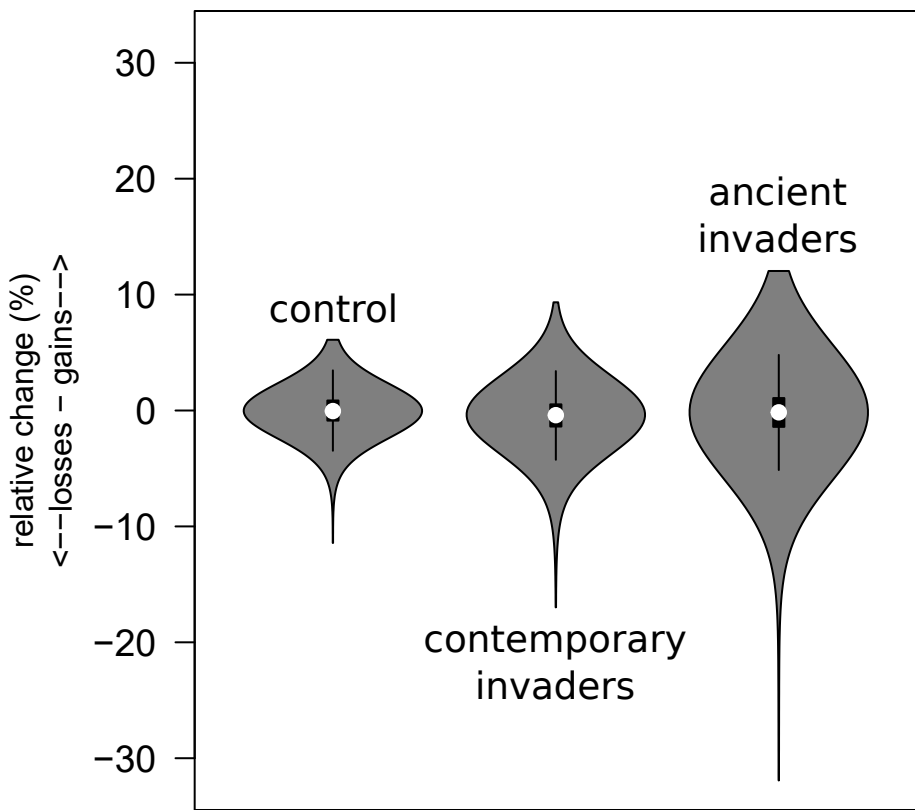

Supplement: S3 Fig — Boxes indicate the 1st and 3rd quartiles, white dots indicate median values and whiskers indicate largest/lowest points inside the range defined by the 1st or 3rd quartile + 1.5 times the interquartile range. (PDF) [file pcbi.1011268.s004.pdf]

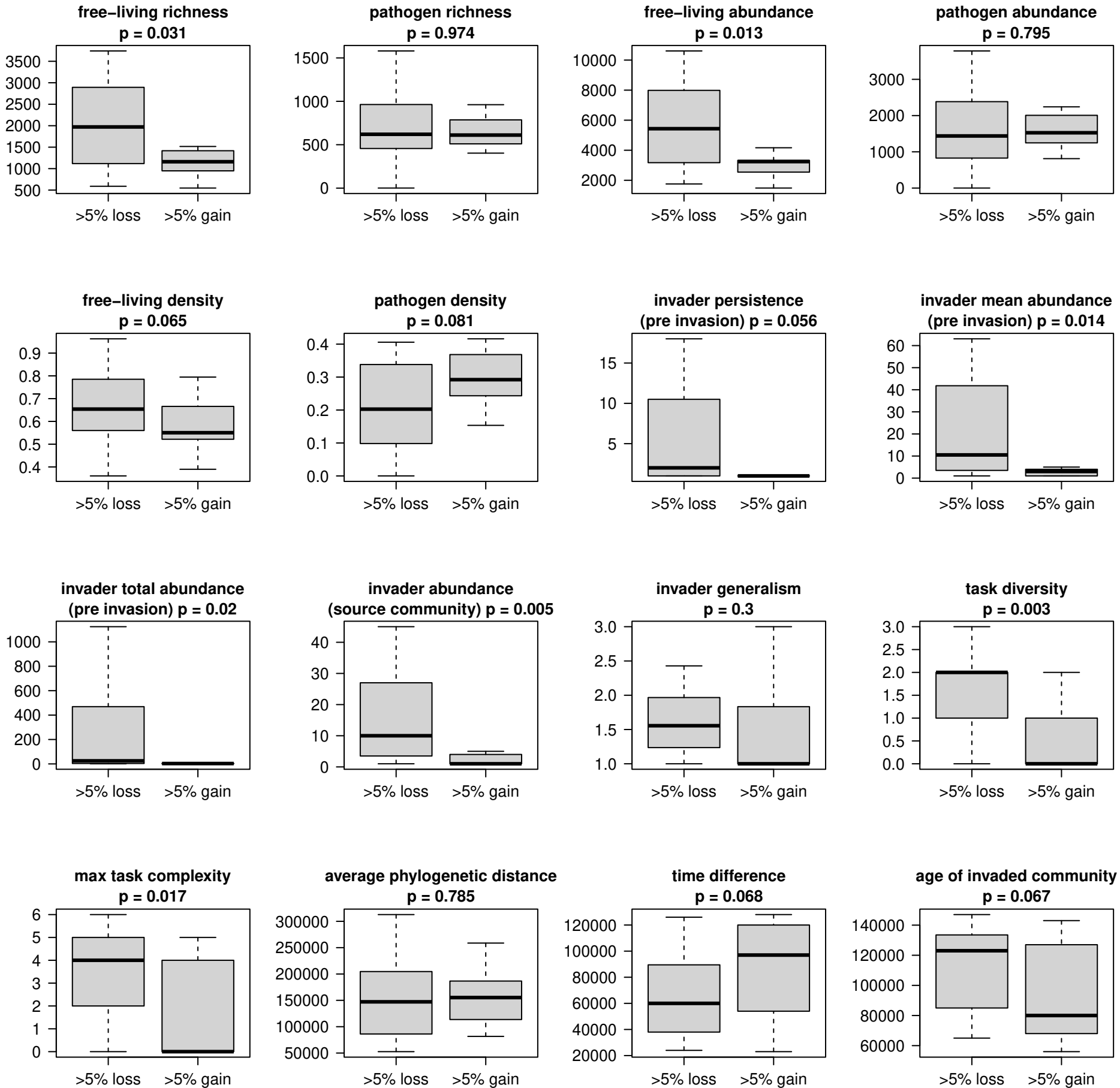

Supplement: S5 Fig — Type I probabilities (p) derived from Wilcoxon double-sided test (nhigh loss = 23; nhigh gain = 13). Boxes indicate the 1st and 3rd quartiles, horizontal lines indicate median values, whiskers indicate largest/lowest points inside the range defined by the 1st or 3rd quartile + 1.5 times the interquartile range. Where not otherwise specified, community properties (e.g., free-living richness, pathogen richness, etc.) refer to the recipient (i.e., invaded) community at the time of the invasion. (PDF) [file pcbi.1011268.s006.pdf]

**invaders' persistence**

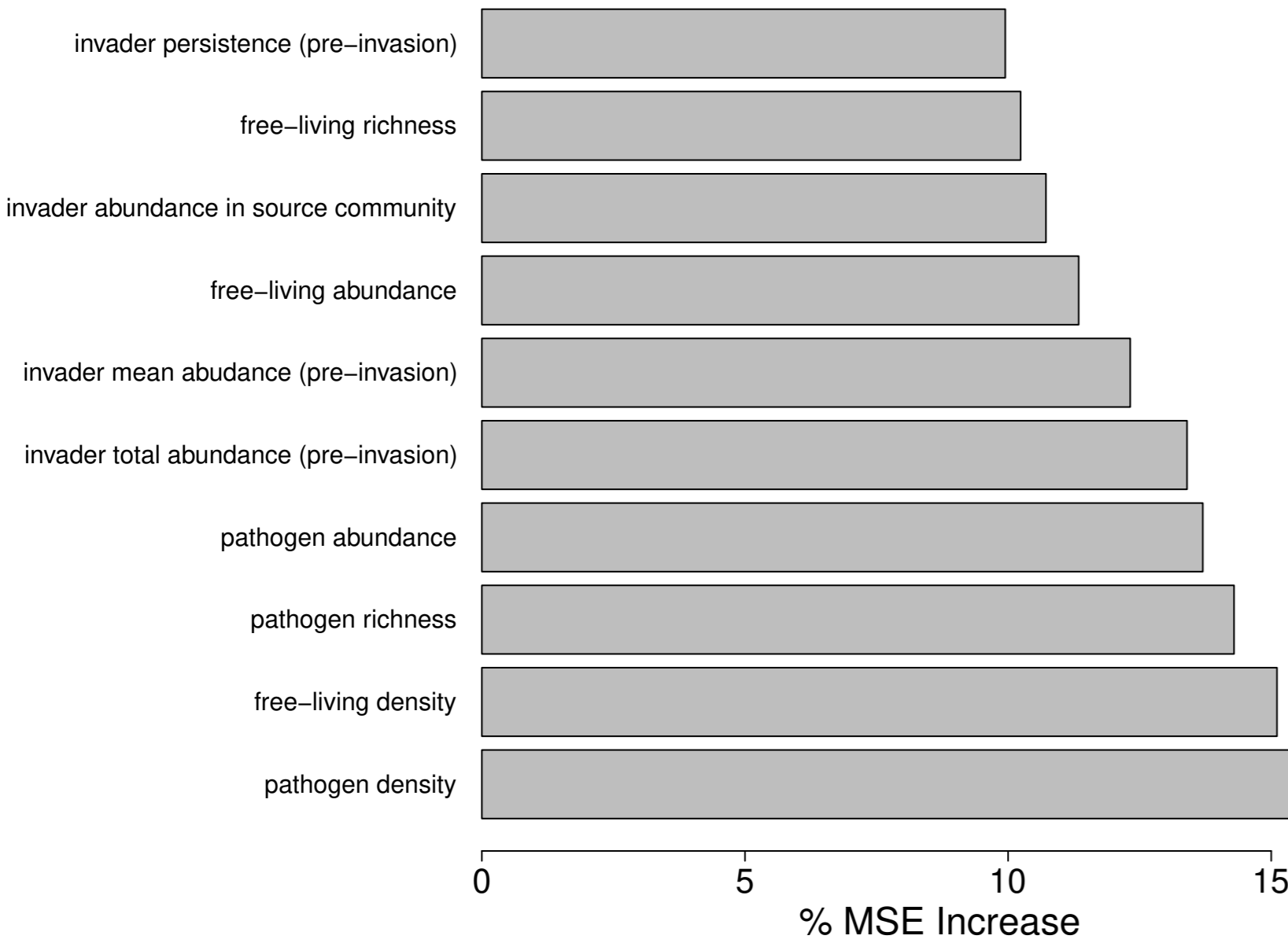

**signed diversity change**

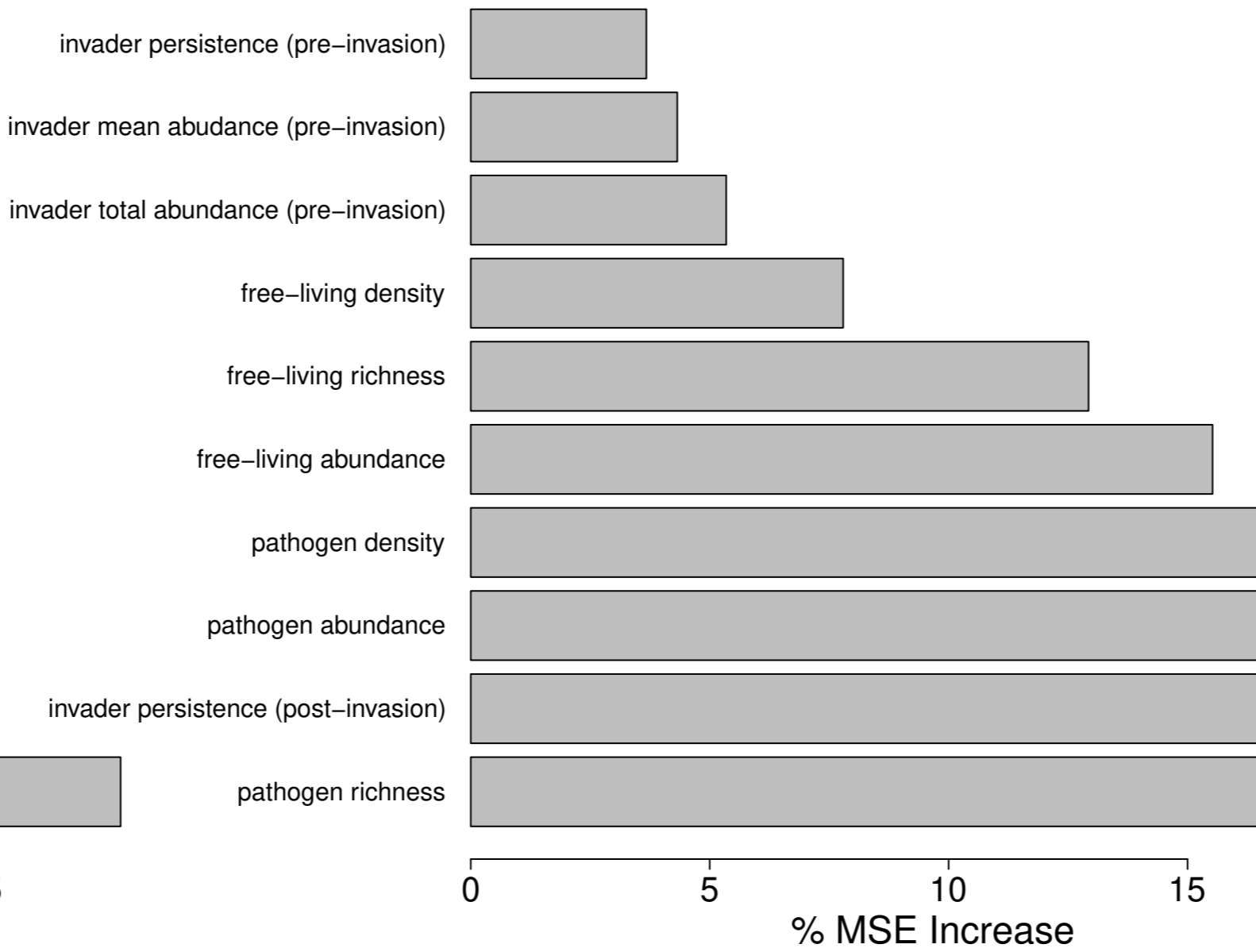

**absolute diversity change**

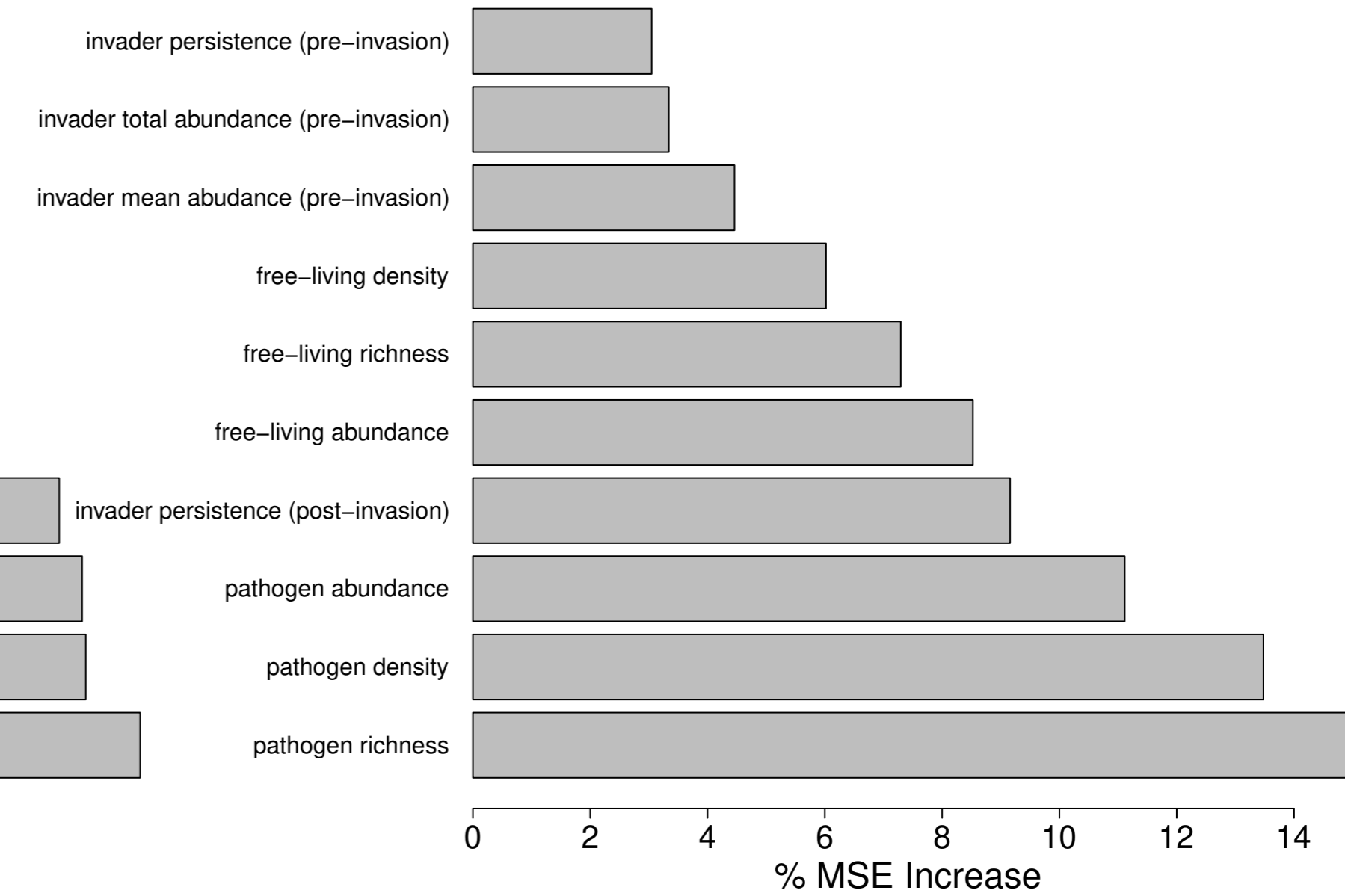

Supplement: S6 Fig — The three models are random forest regressions aimed at predicting time-travelling invaders’ post-invasion persistence (left panel), and relative signed (middle panel) and absolute (right panel) diversity change in invasion versus control simulations based on several features of the invaders and of the invaded communities (see Methods or main text for the full list of considered variables). Variable importance is computed as the percentage increase in the mean squared prediction errors (MSE; estimated through out-of-bag validation) following the random permutation of the target variable. Higher MSE indicate more predictive variables. (PDF) [file pcbi.1011268.s007.pdf]

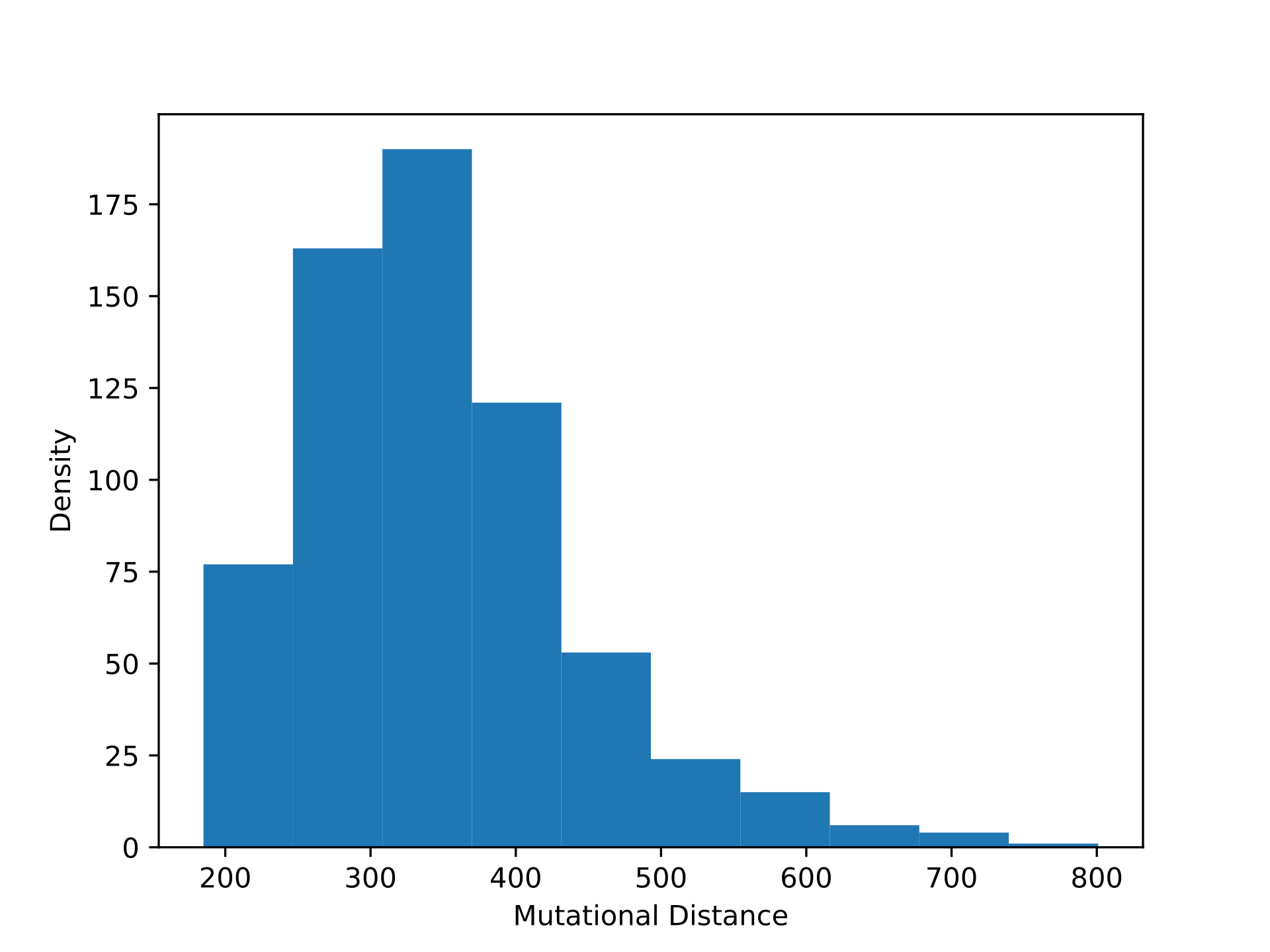

Supplement: S7 Fig — (PNG) [file pcbi.1011268.s008.png]
