# Supplementary material for: Time-travelling pathogens and their risk to ecological communities
Source: PLoS Comput Biol. 2023 Jul 27;19(7):e1011268. doi: 10.1371/journal.pcbi.1011268 (PMC10374110; doi:10.1371/journal.pcbi.1011268)
Supplement: S4 Fig — Signed (a) and absolute (b) percentage change in species richness in invasion simulations compared to control simulations for different values of invader persistence (expressed as the natural logarithm of the number of updates in a simulation). Boxes indicate 1st and 3rd quartiles, horizontal lines indicate median values, whiskers indicate largest/lowest points inside the range defined by the 1st or 3rd quartile + 1.5 times the interquartile range and circles indicate outliers. (PDF) [file pcbi.1011268.s005.pdf]

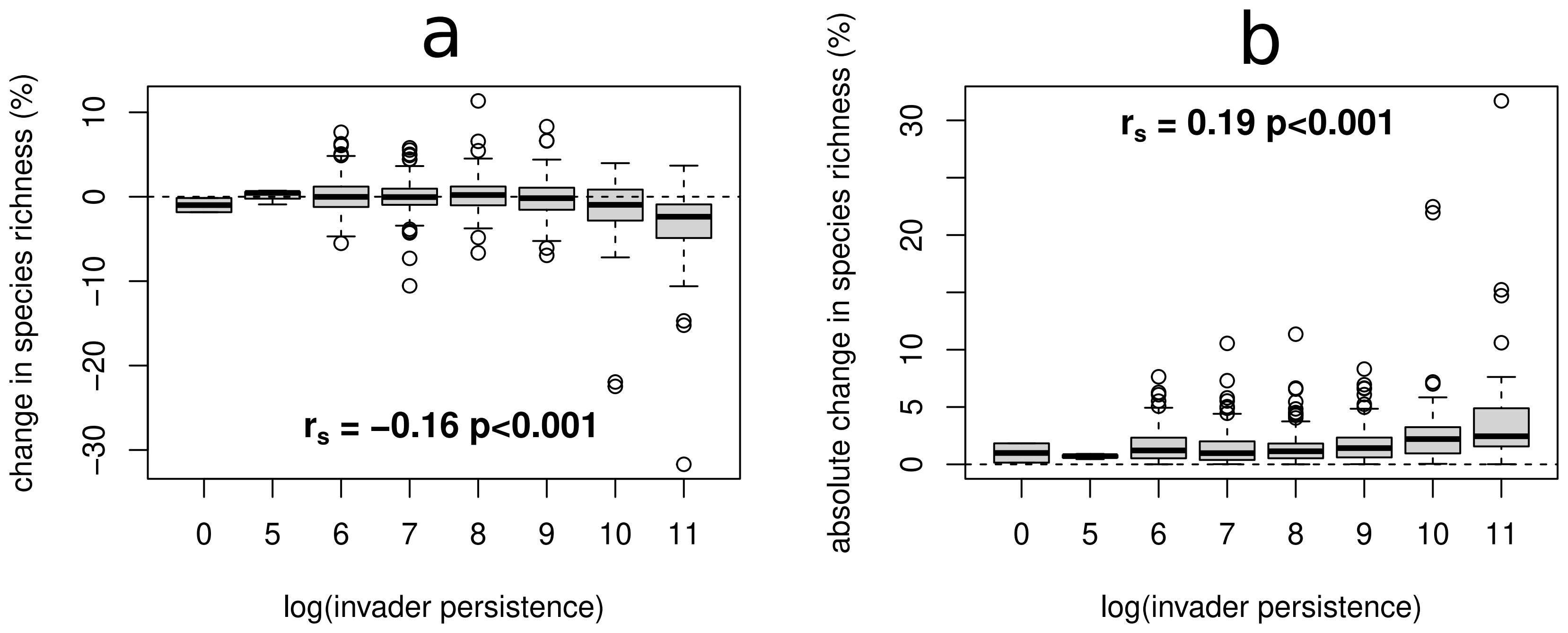
a
change in species richness (%)
10
0
−10
−20
−30
r_s = −0.16 p<0.001
0
5
6
7
8
9
10
11
log(invader persistence)
b
absolute change in species richness (%)
30
20
10
5
0
r_s = 0.19 p<0.001
0
5
6
7
8
9
10
11
log(invader persistence)
