# Supplementary material for: Time-travelling pathogens and their risk to ecological communities
Source: PLoS Comput Biol. 2023 Jul 27;19(7):e1011268. doi: 10.1371/journal.pcbi.1011268 (PMC10374110; doi:10.1371/journal.pcbi.1011268)
Supplement: Attachment — Submitted filename: response_to_reviewers_12_05.docx [file pcbi.1011268.s009.docx]

Dear Editor,

Thank you for the opportunity to resubmit a revised version of our manuscript. Despite the decision of minor revision, we have in fact taken into account the reviewers’ comments, and we have done an extensive set of novel simulations and analyses that provide additional evidence of the robustness of our results, as well as other insights into the phenomenon of emerging pathogens.

Following the suggestions of Reviewer 2, we ran 1100 pairs of simulations comparing controls where we allowed the communities to evolve without any disturbance, with invasion-like mechanisms where the ‘invaders’ consisted of one pathogen randomly selected among the extant ones, and then re-injected into 5% of the grid cells. Those experiments provided us with a proper frame of reference to test whether the ecological effects of time-travelling invasions stemmed from the increase in pathogen density following the injection of a time-travelling invader into the modern community, or from the effects linked to the co-evolutionary separation between the time-travelling invaders and modern communities. These new analyses have confirmed the latter phenomenon, with the range of ecological change caused by ‘contemporary’ invaders having about half the amplitude of that caused by time-travelling invaders.

We have also added a sensitivity analysis where we explored the effect of choosing different time windows for the assessment of ecological change, which supported our initial choice (i.e., 2500 simulation steps from the invasion).

We also added another analysis where we quantified the mutational distance between modern pathogens and their ancestors to draw parallels between the time scale of our simulations and the real world. This revealed that the runtime of our simulations roughly corresponds to 2 million years of bacterial evolution, and that our invaders’ age should be in the order of half a million years. Considering that experiments have shown viability in 750,000 year-old microbes, our enhanced results are now better grounded in real-world analogues. Nevertheless, we have carefully followed the reviewers’ recommendation about being more explicit in the caveats associated with translating our findings to the real world.

We list below our detailed responses to all the reviewers’ queries.

With best regards,

Giovanni Strona

**Reviewer #1**

*The way some of the key features of pathogen dynamics are modeled is either unclear or presented too late in the manuscript. Solving this issue would make the manuscript more convincing to infectious disease biologists.*

*(1) Host specificity: the notion of generalist/specialist is mentioned for the first time on line 207, but the relationship with task complexity is explained only on line 482.*

**Response #1**: We agree. We originally kept most of the description of *Avida* in the Methods section to streamline the manuscript, but we have now added a more detailed section describing the main features of the system in the Introduction (lines 70-112).

“We constructed a large set of artificial evolution experiments where digital virus-like pathogens from the past invade communities of bacteria-like hosts and their contemporary pathogens. For this, we used *Avida*, an artificial-life system simulating *in silico* evolution of complex communities of digital micro-organisms ecologically similar to bacteriophage viruses^21^. The *Avida* world consists of a bi-dimensional grid where sessile digital organisms interact with the environment by performing logical operations, which in turn define their phenotype in functional terms (i.e., phenotypes are defined by the set of logical operations organisms can compute). In the simulated world, organisms compete for CPU cycles—the source of energy permitting them to reproduce—and for space. Time in *Avida* is measured in *updates*, where during each update organisms in the population have each executed an average of 30 single instructions (*i.e.,* 30 CPU cycles). To reproduce, organisms must copy their genetic code, consisting of simple computer instructions, into another location in memory before spawning a new *nearly* identical offspring organism (like what some classes of computer viruses do). While copying themselves, organisms can make errors. Most of the errors are deleterious, resulting in organisms that cannot reproduce. Yet, in some cases, the errors do not affect the new organism’s competitive ability, or can even provide it with a competitive advantage over other organisms. In such cases, the new genotype may engender a higher chance of becoming fixed in the population.

In this way, communities of digital organisms become complex through processes of ‘natural’ selection, starting from a single, viable ancestral genotype. In *Avida*, organisms can have free-living and pathogenic lifestyles (like those of bacteriophage viruses). These lifestyles are distinct, meaning that free-living species cannot become (or evolve into) pathogens and vice versa. Avidian hosts and pathogens are similar in their construction and ‘biology’, but with the difference that the latter cannot draw energy directly from the environment. Instead, pathogens in *Avida* use CPU cycles originally allocated to their hosts, thus reducing their host’s reproductive ability. Pathogens can only survive on suitable hosts and cannot move from one host to another. Therefore, novel infections happen with the transmission of a pathogenic propagule from an infected to a susceptible host (with propagules randomly allocated to grid cells and surviving only if occurring in a grid cell occupied by a suitable host). A given host can be infected by only one pathogen at a time. Pathogens can infect hosts based on a task-matching mechanisms (*i.e.*, for infection to be successful, the infecting pathogen must be able to do at least one of the logical tasks performed by the potential host). Thus, acquiring the ability to do novel tasks (or, in general, tasks uncommonly done by pathogens) can confer an adaptive resistance to hosts, while pathogens can expand their host range and/or increase their prevalence into Avidian communities by evolving the ability to do ‘popular’ tasks. Because the possible logical tasks have different complexity, meaning that they require a different theoretical minimum number of basic code building blocks to be done, and hence pose different evolutionary challenges, host-pathogen dynamics give rise to a co-evolutionary arms race promoting the natural emergence of organismal complexity^22^. The bipartite antagonistic networks derived from these co-evolutionary processes are realistic and match those observed in real-world systems^23,24^. Because, in our experiments, doing tasks requires using some specific quantity of a specific resource, and results in the ‘excretion’ in the environment of by-product resources according to a pre-defined configurable ‘biochemistry’^21^. This means that the task done by the different organisms are both contingent on and affect the environmental conditions, which adds realism to the simulations (*e.g.,* by creating the potential for phenotypic plasticity in Avidians^25^).”

*(2) It is unclear to me how immunity is accounted for. I understand that host can evade pathogens by evolving novel tasks (if I understood well), which represent an innate barrier to infection (e.g., lack of compatibility between a host cell receptor and a virus receptor-binding domain), but I do not understand how adaptive immunity is accounted for. I am particularly thinking about potential cross-reactions between native pathogens and the time-travelling pathogen, as we expect for instance for small pox (the extinct, hopefully not time-travelling, pathogen) and monkey pox (the native pathogen, still circulating in human populations) – see Lloyd-Smith, J. O. (2013). Vacated niches, competitive release and the community ecology of pathogen eradication. Philosophical Transactions of the Royal Society B: Biological Sciences, 368(1623), 20120150. https://doi.org/10.1098/rstb.2012.0150*

**Response #2**: In *Avida*, infection is fully controlled by the task-matching mechanism. Not doing a certain task provides immunity from specific pathogens, so it can be considered a form of adaptive immunity, while hosts possibly do not have any form of innate immunity. We have now mentioned this component, and also added the ‘vacated niche’ to the set of our initial hypotheses (lines 120-126).

“Modern hosts might also have escaped from ancient pathogens during their co-evolutionary history, and possibly retain their evolved resistance, thereby challenging invaders to find susceptible hosts. However, we can envision contrary scenarios where modern hosts have lost this resistance if enough time has passed from their past co-existence with the invader (possibly leaving a ‘vacated niche’ which might be re-occupied by the pathogen if reintroduced^27^). In that case, invaders might have an advantage over modern pathogens actively involved in the ongoing host-pathogen arms race.”

*(3) Please clarify how “transmission” happens. When a host is infected, it sheds the pathogen on its cell grid, and other hosts can get infected if visiting this cell grid and matching with the pathogen task-wise? If that is the case, how long does a pathogen remain infectious on a cell grid?*

**Response #3**: We now clarify the transmission process in the *Avida* overview before the Results (see Response #1). Host and pathogen individuals cannot move from one cell grid to another; however, when an individual (either host or parasite) reproduces, its offspring are allocated to a different cell chosen according to predefined rules (in our set-up, this was a randomly selected cell). A new-born pathogen ending up in an empty cell or in a cell occupied by an immune individual would not survive; by contrast, a new-born pathogen ending up in a cell occupied by a susceptible host would infect that host.

*Related to point (2) above and to the statement made lines 40-41, adaptive immunity is also (in addition to co-adaptation) a key factor in invasion by pathogens (also discussed in Lloyd-Smith, 2013).*

**Response #4**: We now mention this (lines 120-121), even though we preferred to talk about “evolved resistance”, which better reflects the processes happening in *Avida*. See also Response #1.

*How is a “species” (line 126) defined in the model? Is there a threshold of divergence between two individuals to consider they belong to different species?*

**Response #5**: In *Avida* (as for real-world microorganisms), an ‘objective’ identification of species is challenging. We now clarify that we focused conservatively on distinct genotypes as ‘species’ (line 176). As we discussed, the measures we obtained for species/genotype diversity were strongly correlated with those for phenotype and phylogenetic diversity, which supports our choice.

“We explored the ecological effects of time-travelling invaders by comparing the diversity (individual genotype richness and Shannon’s *H* genotype diversity) and abundance of free-living distinct genotypes (e.g., Avidian ‘species’) in recipient communities compared to their control counterparts following invasion.”

*Because the manuscript presents the results before the methods, some terms should be briefly defined earlier:*

*(4) Complexity (line 76)*

*(5) Task (line 190)*

**Response #6**: We now provide definitions for complexity and task in the new Introduction dedicated to *Avida* (lines 75; 102-106; see Response #1).

*Please also include a brief definition of “Shannon’s H evenness”.*

**Response #7**: We now provide the formula for Shannon’s diversity in the Methods (lines 732-734).

“… and Shannon’s diversity of genotypes ($H'=-\sum_{i}^{S} p_{i}log(p_{i})$, with $p_{i}$ = proportion of individuals in the population with the *i*^th^ genotype, and *S* = total number of species in the population)”

*I suggest using “pathogen” instead of “virus” as much as possible in the manuscript as I believe the model could also apply to bacteria and other pathogens, or at least be consistent and avoid switching back and forth between the two words.*

**Response #8**: We have checked the manuscript for consistency, remaining with the term ‘pathogen’. We agree that our results and conclusions might be valid for different classes of pathogens. However, we mention in the introduction that Avidian pathogens are closer to real-world bacteriophage viruses.

*I was also a bit confused by the wording “free-living” (line 26). Could this be called “potential hosts” (e.g., at the first occurrence: “free-living (potential host) species”)?*

**Response #9**: Done.

*Lines 144-… – It is a bit confusing to say “Regardless of invader persistence, invaders had a negligible effect on diversity…”, followed by “However, focusing selectively on successful invasions […], the observed relative loss of free-living species diversity increased to 89.5%”. Could the beginning of the sentence be replaced by “On average” or “overall”?*

**Response #10**: We have modified the sentence as suggested (line 193):

“Overall, invaders had …”

*Line 328 – Add “COVID-19” as there are several pandemics happening at the moment (HIV, avian influenza…).*

**Response #11**: Done (line 423):

“… in light of the recent COVID-19 pandemic”

*Line 301 – “Avida” to be italic?*

**Response #12**: We have now italicized all incidences of *Avida* throughout the text, but when we use the term as an adjective (e.g., “Avidian species”), we have not italicized it (to differentiate the program *Avida* from its [Avidian] constituents).

*Line 490 – Add “grid” in “15,000 [grid] cells” if that is correct, to make it explicit that you are not talking about organism cells.*

**Response #13**: Done.

*Lines 526-528 – Is the ancient anthrax strain still circulating? If not, should not you say that this reemergence event ended up with the death of the pathogen?*

**Response #14**: We have now reworked the paragraph, also to clarify that there is virtually no information available on the potential effects of ancient pathogens on modern communities, and that the only related real-world examples we have refer to the occasional re-emergence of recently disappeared or rare diseases (lines 607-619).

“The potential threats ancient pathogens pose to modern ecological communities are mostly unresolved. The closest empirical examples available are rare and isolated episodes of re-emergence of recent, virtually eradicated diseases^50^. Similar events of reappearance of bygone pathogens often result in severe infections, such as when several people in Siberia were exposed to anthrax spores likely coming from carcasses of infected long-dead animals buried in permafrost, which were released by ice thawing^51^. There could potentially be many unrecorded cases where pathogens severely affected host communities (particularly for bacterial communities analogous to our model agents), as well as undocumented examples where contact between a bygone pathogen and a modern ecological community resulted in either the death of the pathogen, or in its benign re-establishment. However, this knowledge gap, does not prevent the opportunity of formulating and testing *in silico* hypotheses regarding the processes leading to a higher probability of successful establishment in, and the noxious consequences for, recipient communities based on recorded biological invasions.”

**Reviewer #2**

*My main criticism is that some of the caveats should be given more space in the discussion, especially the caveat that right now there isn’t a way to disentangle the confound of parasite invasion and disturbance to the community. Additionally, while simulations are critical to our understanding, the world of AVIDA is so different from our world that it is quite difficult to interpret things like % of successful invasions—we can’t know at the moment whether this has any relationship to real life. My other main criticism is that a number of important details are in the methods which should really go in the introduction, and finally that more effort should be made to situate this paper in the existing invasion simulation literature.*

**Response #15**: Thank you for the constructive comments and suggestions. We have now run an additional set of simulations where we contrasted control scenarios to scenarios where invasions were done by a contemporary pathogen to disentangle the effect of increased pathogen density from that of lack of host-pathogen co-evolution between time-travelling invaders and modern communities. We have also added more caveats regarding the differences between *Avida* and the real world, and we have expanded the Introduction and Discussion to contextualize the paper within the invasion literature.

How does the number of intervening generations in the simulations compare to the estimated number of intervening generations in e.g. melting ice?

**Response #16**: We have now explored this important aspect by looking at the distribution of mutational distances from dominant parasites to the ancestral parasites in our simulations. We found an average of ~ 400 substitutions along the lineage of the most abundant parasite to the ancestor in these runs.

If we make some Fermi calculations and think of a single mutation on a lineage as a single substitution in *E. coli* (a conservative comparison — mutations in *Avida* are much more ‘functional’ than a single nucleotide), our simulations might correspond to ~ 2 million years of total evolution (or at least 800,000 generations in terms of ‘lab’-based mutation rate). Thus, our invaders (sampled from populations between 50,000 and 150,000 simulation steps over a total of 250,000 simulation steps) could come from a real-world equivalent of 0.8 to 1.6 million years ago. Considering that viable bacteria have been recovered from glacial ice > 750,000 years old, our simulations provide a more than reasonable (co)evolutionary time frame. We have now added these values to the Discussion (lines 379-391):

“Although we cannot provide a definitive answer to this question, we can use Fermi estimations incorporating mutational distances between evolved Avidians and their ancestors. We found an average of ~400 substitutions along the lineage of the most abundant parasite to the ancestor in these runs (Fig. S7). If we consider a single fixed mutation along Avidian lineages as equivalent to a single substitution in *Escherichia coli* (a conservative assumption in some respects, considering that mutations in *Avida* are much more ‘functional’ than single nucleotides), and use an estimated 0.0001-0.0002 mutations per generation of *E. coli* to scale our observations, we can estimate that the length of our simulations roughly correspond to ~2-4 million generations of microbial evolution. Thus, our invaders (sampled from populations in between 50,000 and 150,000 updates over a total of 250,000 updates) could come from an approximated equivalent of 400,000 – 2,400,000 bacterial generations. Considering that viable bacteria have been recovered from ~750,000 years old glacial ice^41^ and that natural microbial populations typically have long generation times, our simulations fall within a more than reasonable (co)evolutionary time frame.”

*The colorbar label “steps” in fig 2cd is hard to interpret; the legend says “different relative persistence of invaders” which made me assume it is trying to show the x-axis data from fig 2a, but I may not be following. Please clarify.*

**Response #17**: We have now clarified that the colours indicate the overall persistence of each invader lineage, i.e., the total number of steps that an invader lineage persisted (post-invasion) in the simulations.

*I think Avida could be better introduced in the introduction rather than just in the methods, especially the key difference between free-living and parasitic individuals. Also, can free-living hosts also be parasitic? If so, it should be clarified how this class of organisms was dealt with.*

**Response #18**: We now provide a comprehensive description of the most relevant features of *Avida* in the Introduction, including a more detailed description of Avidian hosts and parasites (lines 70-112; see Response #1).

*I appreciate the goal of exploring many different simulation setups (input-out ratios, world size, etc). But this then begs the question of whether these simulation variables influenced the outcome of invasion. Perhaps these interesting variables could be added to the correlation map in Figure 5?*

**Response #19**: We have now added the ‘environmental’ variables to Figure 5. These are unrelated to relative changes in diversity in invasion *versus* control simulations, so they do not affect (at least directly) the outcome of invasions. See lines 657-661:

“We did not have any specific expectation on the *direct* effects of the environmental variables (world size, abundance and diversity of resources, *i*–*iii*) on the persistence and ecological impacts of invaders on the invaded communities. However, we expected these variables to have potential indirect effects on the outcomes of the invasions by affecting the diversity and complexity of the evolved communities.”

and lines 269-270:

“We found no direct associations between the ‘environmental’ features of the simulated worlds (i.e., world size, abundance, diversity of resources) and the outcomes of the simulations.”

*Why was the invasion on a random step? Doesn’t this make it harder to interpret comparisons at gen 250000, since different simulations have had a different number of generations since invasion?*

**Response #20**: We have now clarified that our choice was motivated by the aim of exploring the effects of different levels of community maturity/complexity on the invasion. That is, we wanted to test whether invasions could have been easier in communities that had evolved for a long time compared to younger communities. This is an important aspect because it relates to the degree of community disturbance and isolation, which could affect the time a given ecosystem has been left undisturbed. The choice does not create any issue for the comparison because we did all our analyses in a standard window centred at the time of invasion.

Our choice of the time intervals for selecting the invaders and for the invasion always gave a comparable window of post-invasion time for unbiased comparisons (i.e., at least 50,000 steps after the invasion, which is the maximum time we considered). For the ‘ecological change’ analyses, we also focused on a much shorter time window (2500 steps; also see also Response #25). See Lines 183-185:

“Therefore, we focused instead on the ecological time scale and compared diversity in the first 2500 updates after invasion (corresponding to 1% of total simulation time).”

*“Hypothesis of the determinants of invader success” seems more like intro or discussion than methods.*

**Response #21**: We have carefully considered this suggestion. Because we have already added a lot of text to the Introduction, we prefer to keep this section in the Methods to avoid overcomplicating the main text. In fact, the hypotheses in this section are specific and to the comparative analysis and do not appear out of place in the Methods. But should the Editor disagree, would be happy to move the section to the Introduction.

*One of the key hypotheses is “if the source and the invaded community have a long temporal separation, the modern hosts might be too complex for the pathogen to proliferate.” Is this possible in AVIDA? From reading the methods, a parasite only needs to be able to do the same task as a “host” to consume its CPU cycles. Testably, what % of invaders matched at least one host’s task at the moment of invasion? This would help disentangle failures due to lack of niche from failures due to intra-pathogenic competition.*

**Response #22**: We now clarify that the task-matching criterion refers to the tasks done by each organism at a given moment that depend in turn on many different conditions. Those conditions are specific to a given organism and related to the availability of resources that is itself determined by a complex combination of the resource inflow-outflow and of the tasks done by the other organisms. In fact, doing a given task requires using some amount of a given resource and results in some by-product (which could also be a different resource from the one used by the organism).

The rules determining the association between resources and tasks are pre-defined in what could be considered the basic biochemistry of the simulated system. Identifying ‘reactions’ where a given amount of a specific input resource is required to do a given task/logical operation might possibly also return or transform part of the resource. Depending on the number and complexity of these reactions, unexpected ecological mechanisms can emerge (e.g., generating ‘trophic’ dependencies between organisms; doi:10.1007/978-3-642-30425-5_3, Fig. 3.2). In our experiments, we determined the energetic reactions randomly for each simulation (following Strona and Lafferty 2016) to represent a broad range of systems with varying potential ecological structure.

All of this it is to say that only looking at the potential tasks done by hosts and parasites (e.g., the set of instructions provided by their genotype) might be deceiving, and not useful to “… disentangle failures due to lack of niche from failures due to intra-pathogenic competition”. In principle, considering that in all simulations all tasks are potentially done/do-able by some extant species, what matters is how often those tasks are done and matched by a pathogen.

However, we have now expanded the analyses and reported a more informative measure, which is the generalism/host range of the invaders after the invasion. This tells us how many different hosts the pathogen could exploit in the modern community. That measure (see pairwise correlations; Fig. 5) is positively correlated with the original generalism of the pathogen in its native community and, not surprisingly, with the pathogen’s persistence in the invaded community. This suggest that being compatible with many hosts is fundamental for successful establishment. This positive effect interacts with the negative effect due to pathogen’s competition, but it is difficult to disentangle the individual effects from their potential interactions. See lines 278-283:

“Invaders that were generalist in the source (ancient) communities (i.e. capable of using many different hosts) also tended to be more persistent (*r_s_* = 0.24). This is consistent with the relationship observed for task diversity, which contributes to generalism due to the task-matching criteria determining pathogen infection in *Avida*. In turn, highly persistent invaders tended to use a larger variety of hosts after their reintroduction into future communities (*r_s_* = 0.70).”

*I think the no-invasion control was a good choice. Another choice could have been to do a “local” (or “modern”) invasion, where 5% of the world’s cells received extant pathogens (this seems like what the “natural” variation controls was aiming at, but didn’t quite capture). The current control leaves invader presence and change in density confounded, my suggested control would remove the density confound. I don’t think this is necessary but if not done, the confounded nature of invasion and change in density should be remarked upon as a caveat in the discussion. Put more generally, right now it is hard to pin down the effects on diversity to a cause of the specific invader, or the fact that there was generally “disturbance.”*

**Response #23**: Good suggestion. We have now run a new set of control simulations where we randomly selected a pathogen from the modern community and re-injected it in 5% of the simulation cells, hence simulating the same perturbation as that generated by the time-travelling invaders. We then compared the change in those control simulations with that observed in the time-travelling invasions. As it was for the no-invasion controls, the disturbance following the re-injection of a modern pathogen did not generate the ecological change that we observed in the time-travelling invasions. This novel analysis confirms that the time/evolutionary separation between invading pathogens from the past and the modern communities is the true determinant of the ecological impacts, not just the momentary increase in pathogen abundance following the appearance of a pathogen. See lines 204-228 and Fig. S3:

“**Diversity changes in time-travelling invasion simulations *versus* background expectation**

Differences in diversity trajectories between control and invasion simulations might not necessarily imply a direct effect of invading pathogens on control communities. In fact, we cannot exclude *a priori* that running independent, alternative replicates of a community’s future from a given point in time would produce comparable differences even without selective manipulation (e.g., the injection of time-travelling invaders). To rule out this potential problem, we ran an additional set of paired simulations where we did not translocate the invader at time of invasion. That is, instead of having paired control-invasion simulations, we ran paired control-control simulations. For these, we applied the same settings of the main experiment, and then randomly reduced the set of simulations to match the number of replicated pairs in the main simulations (*n* = 748, after removing simulations where pathogens went extinct before the invasion; see Methods). By contrasting two alternative futures of the same control simulations, we could quantify the expected range of variation in free-living species diversity in the absence of external disturbances (i.e., time-travelling invasions). The direction of diversity change in the control-control simulations is necessarily arbitrary. In our experiments, we identified the reference simulation in each pair *a priori* (i.e., before each run).

The control-control experiment provided strong support for the primary role of invasions as drivers of diversity change. In fact, the range of variation in average diversity change between control-invasion simulations was 2.5 times greater than that observed in control-control simulations, with relative change in the latter varying from 11.4% loss to 6.11% gain (Fig. S3).

In 31.6% of the highly persistent invasions, the relative diversity loss was greater than the upper 95^th^ percentile of the relative diversity change observed in the control-control simulations, indicating strong statistical support for the hypothesis that the diversity changes observed in the invasion simulations cannot be explained by the expected stochastic variation in replicate community dynamics.”

*“we limited the comparison to simulations where there was at least one native pathogen with a population size N at least as large as the invader’s,” <- this is interesting and your rationale about pop size makes sense, but it does change the scope of the experiment, because it is biasing towards worlds where we know that at least some pathogens can exist. Important caveat.*

**Response #24**: This is important. Our approach is in fact consistent with the suggestion on the additional control experiment with the ‘invasion’ of extant pathogens. In our initial data exploration/analyses, we had kept the simulations where time-travelling invasions happened in systems without pathogens (i.e., where pathogens went extinct naturally in Avida before the invasion). The results revealed a much stronger effect of time-travelling pathogens, but we realized that the comparison was unfair and possibly misleading because it was also showing the generic effect of pathogens on host communities, and the specific effects stemming from the fact that those pathogens came from the past. The first effect likely overshadows the latter; furthermore, it is extremely unlikely to envision a real system without pathogens. The extinction of pathogens in *Avida* should be, to some extent, considered an artefact due to the system being closed to immigration and because pathogens cannot naturally evolve from free-living species.

*“We focused on the ecological time scale here, and hence compared diversity in the first 2500 steps after the invasion” please explain why 2500 was chosen specifically. Would the results have differed much with a 1000 or 5000 step window, for example?*

**Response #25**: Our choice of time interval for the comparison is arbitrary by necessity. We chose 2500 because this was 1% of the total number of steps. We have now explored how imposing shorter and longer windows (from 500 to 5000 steps) affects our results, and they are consistent (see lines 189-192 and Fig. S2).

“We also explored whether the choice of the ecological time frame affected the results by replicating our analyses for different post-invasion windows with sizes spanning 500 to 5000 steps. Because the results were robust to this choice (Fig. S2), we report only the results for the 2500-step window.”

*I appreciate this caveat in the discussion: “Although Avida has proven to be capable of remarkable realism in simulating evolutionary and ecological processes34–37, it is clear that there are substantial differences between Avidians and real world microorganisms.” I think it could go a bit further though, because as it is, the lack of similarity means that I’m not sure we should really give much interpretation to the 3% success.*

**Response #26**: We have now expanded the section to clarify/emphasize that our findings cannot necessarily be translated directly to the real world (lines 373-379, and also response #16).

“Although *Avida* has proven to be capable of remarkable realism in simulating evolutionary and ecological processes^23,24,39,40^, it is clear that there are fundamental differences between Avidian digital worlds and our own. One important aspect is that simulation updates and generations in *Avida* cannot be directly translated into a consistent measure of time, which might cast doubt on how our experiments aligns with real-world settings. In other words, how does the number of updates in the simulations compare to the estimated number of intervening generations in melting ice, for example?”

*While this may be the first sim to specifically examine “time-traveling” invasions, there is a wealth of existing literature simulating invasion generally. More reference to existing work would be place this article in the existing knowledgebase.*

**Response #27**: We have now expanded the references to extant invasion literature to contextualize our work better.
